# Salivary levels of TNF-α in patients with recurrent aphthous stomatitis: A cross-sectional study

**DOI:** 10.15171/joddd.2018.007

**Published:** 2018-03-14

**Authors:** Kanad Chaudhuri, Keerthi Krishnankutty Nair, Lingappa Ashok

**Affiliations:** ^1^Former Postgraduate Student, Department of Oral Medicine and Radiology, Bapuji Dental College and Hospital, Davangere, Karnataka, India; ^2^Former Postgraduate Student, Department of Oral Medicine and Radiology, Bapuji Dental College and Hospital, Davangere, Karnataka, India; ^3^Professor and HOD, Department of Oral Medicine and Radiology, Bapuji Dental College and Hospital, Davangere, Karnataka, India

**Keywords:** cytokines, canker sores, TNFα, saliva

## Abstract

***Background.*** Recurrent aphthous stomatitis (RAS) is a disorder characterized by recurring ulcers involving the oral mucosa in patients with no other signs of disease. The current concept of etiopathogenesis is that RAS is a clinical syndrome with several possible etiologies. The process seen in RAS is probably initiated through an as yet unidentified antigenic stimulation of the mucosal keratinocytes, which stimulates secretion of T-cell activation cytokines ‒ interleukins and tumor necrosis factor alpha (TNF-α). TNF-α causes inflammation by its effect on endothelial cell adhesion and neutrophil chemotaxis. The rele-vance of TNF-α to the pathogenesis of RAS has stemmed from the observations that anti- TNF-α drugs such as thalidomide and pentoxifylline have been found to be effective in the treatment of RAS. Therefore, the present study was an attempt to measure the levels of salivary TNF-α in patients with RAS, which will reflect the local production of cytokines at the site of the disease. The aim was to evaluate the salivary levels of TNF-α in patients with recurrent aphthous stomatitis.

***Methods.*** The study comprised of 60 subjects, of whom 30 clinically proven RAS patients of either sex were selected as cases and 30 healthy, age- and gender- matched subjects were selected as controls. After taking informed consent, 5 mL of unstimulated saliva were collected from both the study and control group subjects. Determination of salivary TNF-α levels was carried out by Enzyme-Linked Immunosorbent Assay (ELISA) and expressed in pg/mL. Statistical analysis of the RAS and control groups was carried out using unpaired t-test. Gender-wise comparison of salivary TNF-α levels in the study and control groups was carried out using one-way ANOVA.

***Results.*** Mean salivary TNF-α levels were significantly higher in the RAS group compared to the control group (P<0.001). It was also revealed that the mean salivary TNF-α levels in females were significantly higher than in males in the study group (PP<0.05).

***Conclusion.*** It is fair to suggest that TNF-α plays a very important mediatory role in the pathogenesis of RAS and may play an important role in the search for a definitive treatment for the disease.

## Introduction

Recurrent aphthous stomatitis is a common condition which is characterized by multiple recurrent small, round or ovoid ulcers with circumscribed margins, erythematous haloes, and yellow to greyish floors, appearing first in childhood or adolescence.^1^

The ulcerative process in RAS is initiated by an unknown antigenic stimulus of the mucosal keratinocytes, leading to T lymphocytic stimulation, the liberation of cytokines such as TNF-α and other interleukins, and the migration of lymphocytes, neutrophils and Langerhans cells.^2^ TNF-α can also stimulate the expression of MHC class I and II antigens in epithelial basal cells. These cells are recognized by T lymphocytes which trigger a cytotoxic response and cause the ulceration.^2^ The aim of this study was to estimate the salivary TNF-α levels in RAS patients prior to definitive therapy.

## Materials and Methods

A cross-sectional study was conducted in the Department of Oral Medicine and Radiology, Bapuji Dental College & Hospital, Davangere, after obtaining approval from the institutional ethics committee. The time period of the study was 1 year. Based on the inclusion and exclusion criteria a total of 60 subjects were included in the study.

Sample size (n) was determined using the following formula:

$$n=\frac{2t^{2}xs^{2}}{d^{2}}$$

$$n=\frac{2{\left(2.13\right)}^{2}x{\left(12\right)}^{2}}{{\left(7.1\right)}^{2}}$$

n=26 in cases and controls

n≅ 30 cases and controls representing normal distribution.

[t^2^= theoretical value of distribution, s = pooled standard deviation, d = mean expected difference (i.e. mean expected difference in TNF-α levels between in subjects and controls)]

Thirty clinically proven RAS patients of either sex were selected as the study group and 30 healthy age- and gender-matched subjects were selected as the control group. Patients clinically diagnosed with recurrent aphthous stomatitis were classified under the types given by Stanley HR.^3^ Only those patients who gave a signed informed consent form voluntarily were allowed to participate in the present study.

Patients with aggressive and chronic periodontitis, habit of tobacco and alcohol use, significant local and systemic diseases and with a history of steroid therapy 1 month prior to the investigation were excluded.

Under aseptic conditions, 5 mL of whole unstimulated saliva were collected into sterilized polystyrene tubes for 10 minutes by passive drool method,^4^ between 9 am and 12 pm, after one hour or more since the last food intake. After cooling for 10 minutes, the samples were centrifuged at 4000 rpm for 10 minutes; the upper parts were drawn and stored in small aliquots at –80°C. TNF-α determination was carried out employing immunoassay and quimioluminiscence techniques^5^ using Boster’s human TNF-α ELISA kit (Bosterbiological Co. Ltd, CA, USA). The salivary samples were cultivated in the presence of specific anti-TNF-α antigen after a controlled defrost and centrifugation. The levels of TNF-α were determined with an automatic analysis unit LISA PLUS (Aspen Diagnostics Pvt. Ltd, New Delhi, India) after the application of a quimioluminiscent substrate.

### Statistical analysis

Data was analyzed by using SPSS 18, with unpaired t-test and one-way ANOVA.

## Results

The study consisted of a total of 60 subjects which comprised of 30 clinically proven RAS patients and 30 controls. Both groups comprised of 19 (63.3%) females and 11 (37.7%) males. The age of the subjects ranged from 18 to 40 years with a mean age of 25.77 years (Table 1). Based on clinical examinations, RAS was classified under the criteria given by Stanley HR.^3^ It was observed that 29 (97%) patients had minor form of RAS and 1 (3%) patient had major form of RAS. No patient had herpetiform type of RAS. The most common sites of involvement of the ulcers were the buccal mucosa, labial mucosa, tongue, vestibule, palate, alveolar mucosa and a combination of one or more sites (Table 2).

**Table 1:** Age-wise distribution of the study and control groups

**Age (years)**	**Case group (%)**	**Control group (%)**
11‒20	6 (20)	6 (20)
21‒30	17 (56.7)	17 (56.7)
31‒40	7 (23.3)	7 (23.3)
Mean ± SD	25.77±6.13	25.77±6.13

**Table 2:** Distribution of cases according to the site of involvement (single site/combination) of RAS

**SITE**	**NUMBER OF CASES (%)**
Buccal mucosa	20 (66.67%)
Labial mucosa	16 (53.33%)
Tongue	14 (46.67%)
Vestibule	2 (6.67%)
Palate	2 (6.67%)
Alveolar mucosa	1 (3.33%)

The mean value of salivary TNF-α was 86.30 pg/mL for RAS cases, whereas in controls the mean value of salivary TNF-α was 47.85 pg/mL. Unpaired t-test was used to check the difference in mean between the two groups. There was a highly significant difference (P<0.001) in the mean salivary TNF-α levels between the two groups (Table 3).

**Table 3:** Comparison of salivary TNF-α levels in study and control groups

	**Salivary TNF-α levels in pg/mL**		
	**Range**	**Mean**	**S.D**
**Case group**	38.7‒121	86.30	18.59
**Control group**	13.3‒72.8	47.85	17.48

t = 8.253 P<0.001 (unpaired t-test)

It was observed that the mean salivary TNF-α levels in males in the RAS group was 76.74 pg/mL, whereas in females it was 91.83 pg/mL. One-way ANOVA was used to check the difference in the mean between the two groups. There was a statistically significant difference in the mean salivary TNF-α levels between the two groups (P<0.05). However, no such difference was seen in the control group (Table 4).

**Table 4:** Gender-wise comparison of salivary TNF-α levels in the study and control groups

**Study Groups**	**Gender**	**Mean TNF-α in pg/mL**	**S.D**	**t**	**P-value**
**Cases**	Males	76.74	19.40	2.296	P=0.029 (Significant)
	Females	91.83	16.10		
**Controls**	Males	45.86	19.60	0.469	P=0.642 (Not significant)
	Females	49.01	16.59		

One-way ANOVA

## Discussion

Recurrent aphthous stomatitis, also known as recurrent aphthous ulcers or canker sores, is among the most common oral mucosal lesions the oral physicians and dentists observe. RAS is a disorder of unknown etiology that may cause significant morbidity. One or several discrete, shallow, painful ulcers are present on the unattached oral mucous membranes. Minor aphthous ulcers typically last 7‒10 days and heal without scarring. Major aphthae may last several weeks to months and can scar when healing.^6^

The immune-pathogenesis of RAS probably involves a cell-mediated immune response mechanism which causes generation of T cells and TNF-α by macrophages and mast cells.^7^ The TNF-α cytokine, a major inflammatory mediator, induces initiation of the inflammatory process by its effect on endothelial cell adhesion and a chemotactic effect on neutrophils.^7^ The primary role of TNF-α is in the regulation of immune cells. Studies have shown that RAS can be prevented by treatments that prevent the synthesis of endogenous TNF-α such as pentoxifylline and thalidomide.^7,8^

Elevated levels of interleukin-2 (IL-2) as well as those of TNF-α (both proinflammatory cytokines) and lower levels of IL-10 (an antiinflammatory cytokine) have been reported in the lesions of RAS patients. IL-10 usually stimulates epithelial proliferation in a healing process; therefore, its low levels in RAS patients may delay epithelialization and prolong the duration of the ulcers.^9-11^

TNF-α also has some important immune regulatory activities, including stimulation of class I major histocompatibility (MHC) expression. An increase in class I and class II MHC antigen expression has been detected in the basal epithelial cells in the pre-ulcerative and ulcerative stages of the RAS lesion.^12^ As almost no MHC antigens were detected after healing, they probably play a role in the local tissue damage by targeting these cells for attack by cytotoxic T-cells (CD8^+^ cells) in the ulcerative process.^12^

In the present study, comparison between the study and control group showed a statistically significant (P<0.001) increase in the mean TNF-α levels in the RAS group compared to the control group. This indicates an increase in the cytokine level during the active stage of the disease. Similar findings have been reported by Eguia-del Valle et al (2011), Batool Al-Ghurabei et al (2011), Boras et al (2006) and Natah et al (2000).^2,5,13,14^ No studies contradicting the above findings have been reported in the literature.

This study also showed a statistically significant (P<0.05) increase in the salivary TNF-α levels in females compared to males in the RAS group. Interestingly there is no literature available which explains such difference. It may be because of the local or systemic and genetic factors that determine this gender discrepancy in TNF-α levels for RAS. However, there was no significant difference in the TNF-α levels between males and females in the control group.

It is fair to suggest that TNF-α plays a very important mediatory role in the pathogenesis of RAS, as the patients with active form of the disease showed a greater elevation of TNF-α in both serum and saliva and these findings have been supported by the observations that Thalidomide, which reduces the activity of TNF-α by accelerating the degradation of messenger RNA, pentoxifylline, which inhibits TNF-α production, and levimasole, which modulates serum TNF-α production, have been found to be effective in the treatment of RAS.^15-17^ The development of a new class of drugs specifically designed to inhibit TNF-α production could be a new approach in the treatment of RAS, with a higher efficacy and safety compared with those drugs available commercially today. A range for salivary TNF-α in normal individuals is yet to be determined but considering that the collection of saliva is a cheap, easy and non-invasive method, further research is sure to provide a positive outcome regarding the role of salivary TNF-α in the pathogenesis and management of RAS.

### Limitations

The findings of this study need to be carefully interpreted because of the small sample size and to the best of our knowledge, lack of sufficient studies involving the estimation of salivary levels of TNF-α in patients with RAS. Further research involving larger samples is suggested along with extensive work involving the specificity of estimation techniques of salivary TNF-α.

## Conclusion

Our study demonstrated a highly significant increase in the salivary levels of TNF-α in RAS patients compared to controls. We conclude that the estimation of salivary TNF-α levels can be used as a reliable biomarker for the progression and retrogression of RAS. All these data support the importance of TNF-α in the pathogenesis of RAS. Further research needs to be undertaken regarding the role of anti-TNF-α drugs in the treatment of RAS, which may lead to the prevention and cure of the disease in the future.

## Acknowledgment

None.

## Conflict of Interests

The authors declare that the there are no conflicts of interest.
